# Evaluating the responsiveness of the Warwick Edinburgh Mental Well-Being Scale (WEMWBS): Group and individual level analysis

**DOI:** 10.1186/1477-7525-10-156

**Published:** 2012-12-27

**Authors:** Hendramoorthy Maheswaran, Scott Weich, John Powell, Sarah Stewart-Brown

**Affiliations:** 1 Division of Health Sciences, Warwick Medical School, University of Warwick, Gibbet Hill Campus, Coventry, CV4 7AL, UK; 2 Division of Mental Health and Wellbeing, Warwick Medical School, University of Warwick, Gibbet Hill Campus, Coventry, CV4 7AL, UK; 3 Department of Primary Care Health Sciences, University of Oxford, Oxford, United Kingdom

**Keywords:** Well-being, WEMBWBS, Responsiveness, Sensitivity to change

## Abstract

**Background:**

Mental well-being now features prominently in UK and international health policy. However, progress has been hampered by lack of valid measures that are responsive to change. The objective of this study was to evaluate the responsiveness of the Warwick Edinburgh Mental Well-being Scale (WEMWBS) at both the individual and group level.

**Methods:**

Secondary analysis of twelve different interventional studies undertaken in different populations using WEMWBS as an outcome measure. Standardised response mean (SRM), probability of change statistic (P̂) and standard error of measurement (SEM) were used to evaluate whether WEMWBS detected statistically important changes at the group and individual level, respectively.

**Results:**

Mean change in WEMWBS score ranged from −0.6 to 10.6. SRM ranged from −0.10 (95% CI: -0.35, 0.15) to 1.35 (95% CI: 1.06, 1.64). In 9/12 studies the lower limit of the 95% CI for P̂ was greater than 0.5, denoting responsiveness. SEM ranged from 2.4 to 3.1 units, and at the threshold 2.77 SEM, WEMWBS detected important improvement in at least 12.8% to 45.7% of participants (lower limit of 95% CI>5.0%).

**Conclusions:**

WEMWBS is responsive to changes occurring in a wide range of mental health interventions undertaken in different populations. It offers a secure base for research and development in this rapidly evolving field. Further research using external criteria of change is warranted.

## Introduction

Positive mental health and well-being has been of interest to philosophers, social scientists and psychologists for some time. Because of growing awareness of its public health impact [1-6] mental well-being is gradually moving towards centre stage, both in UK and international health policy [7,8]. Economists and politicians have also suggested that a ‘shift in emphasis from measuring economic production to measuring well-being’ is called for because of its potential impact on economic prosperity as well as health [9]. While debate surrounds the precise definitions of both well-being and mental well-being [10,11], there is broad consensus that the latter is more than the absence of mental illness and that it covers both eudaimonic (psychological functioning) and hedonic (affective) dimensions [12]. This emerging conceptual clarity has led to the development of a number of candidate measures in the research literature.

The Warwick-Edinburgh Mental Well-being Scale (WEMWBS) was developed to measure positive mental health at the population level. Initial psychometric testing showed that the scale was valid, reliable and acceptable in adult populations across Europe [13-16], in adolescents (13–15 years) [17], and in minority ethnic groups [18]. However, a key property of any measure is its responsiveness or sensitivity to change [19]. This is essential for investigating causal pathways, evaluating interventions and assessing the impact of policy at national level. Responsiveness has not yet been established for measures of mental well-being.

Responsive instruments should have the ability to detect changes in health status where these occur [19]. Several approaches are advocated to evaluate responsiveness [20-22]. Broadly, these are classified as either anchor-based, where an external criterion of change is used to compare the observed changes in scores, or distribution-based, where the observed change is compared to the statistical properties of the sample or instrument [20]. We opted for the latter as our data precluded the former. The literature differentiates the distribution-based methods into whether the instrument is able to detect statistically important changes at the group level or at the individual level [20]. The rationale for undertaking analysis at the individual level is that important changes at the group level may not translate into important changes at the individual level [22]. The objective of this study was to evaluate the wider applicability of WEMWBS. First, we use a combination of both group and individual level measures to evaluate the responsiveness of WEMWBS in a variety of studies undertaken in different populations; and, second, we sought to investigate what change in WEMWBS score may constitute an important change. Our findings provide guidance to investigators using WEMWBS to evaluate the impact of interventions aimed at improving mental well-being.

## Methods

### Study design

We undertook secondary analysis of data collected by registered users of WEMWBS. This instrument is available free of charge, but prospective users are asked to register their intended use with the originator of the measure (Sarah Stewart-Brown). An email was sent with a brief questionnaire to all those who had registered to use WEMWBS by asking for a description of their study. Those who replied and who had used one of these measures to evaluate an intervention were contacted. All data comes from users who had registered with the database from November 2007, when the initial validation study of WEMWBS was published [13], to October 2010. During this period 209 groups had registered interest, of these, 34 (16%) users replied to the email with a description of their study. 15 of the 34 studies used WEMWBS in a before and after study design, the majority (12/15) to evaluate a service development, and few studies (3/15) included a control group. We included all studies irrespective of the type of intervention or population studied, but excluded studies with samples of less than 30 participants (3/15), providing data from 12 studies for analysis. We requested anonymised data together with information about the intervention. Few studies (3/12) collected socio-demographic data on participants and this was therefore not investigated. We used secondary data with no participant identifiers, and permission was granted from the lead investigators of the included projects.

### The Warwick-Edinburgh Mental Well-being Scale (WEMWBS)

WEMWBS is a 14-item scale; each answered on a 1 to 5 Likert scale. Items cover different aspects of eudaimonic and hedonic mental wellbeing and are worded positively. Item scores are summed to produce a total score ranging from a minimum of 14 to a maximum of 70, with higher scores representing higher levels of mental well-being.

### Data analysis

Data were imported and analysed in PASW Statistics 18 (SPSS, Chicago, IL, USA), and a user built program was used to display the data as forest plots [23]. Descriptive statistics were used to assess data quality. As we were evaluating the instrument and not the interventions we assumed that data was missing at random [24] and undertook a complete case analysis. We examined floor and ceiling effects in baseline WEMWBS scores. Instruments exhibit floor or ceiling effects if more than 15% of participants record the lowest or highest score, respectively [25]. Mean and standard deviations of baseline scores were calculated for each study. Normality was verified using the Shapiro–Wilk test and through visual examination of histograms with normal curve, and normality plots.

#### Group level analysis

For all the studies we assumed there had been an improvement in mental well-being and investigated whether WEMWBS was able to detect this. We used the standardised response mean (SRM) [26] to evaluate whether WEMWBS was responsive to change at the group-level in each of the studies. The SRM was calculated by dividing the mean change in score by the standard deviation (SD) of the change score [26], and 95% confidence intervals were constructed by assuming a normal distribution [27]. Although a number of group-level statistics have been used in the literature to evaluate responsiveness [22,28], the SRM is considered the most appropriate when evaluating responsiveness in single group pre-post studies [27,29]. SRM was interpreted by calculating the probability of change statistic P^^^, which represents the cumulative normal distribution function of the derived SRM. The P^^^ statistic denotes the probability the instrument detects a change and ranges from 0.5 (no ability to detect change) to 1 (perfect ability to detect change) [27], 95% CI was estimated using the substitution method [30].

#### Individual level analysis

At the individual level, any change will consist of true change and the error associated with measurement of the phenomenon in question [20]. A commonly used approach estimates the standard error of measurement (SEM) of the instrument [31] and uses this score to determine whether a statistically important change has been detected at the level of the individual. There is no clear consensus of how much greater than the SEM a change needs to be considered ‘true’ (statistically), and a variety of thresholds based on the SEM have been proposed ranging from 1 SEM to 2.77 SEM [22]. As our primary objective was to evaluate whether WEMWBS detected statistically important changes at the individual level, we calculated the proportion of participants (and 95% CI) within each study that would be classified by the instrument as having improved at the higher threshold of 2.77 SEM. A change greater than 2.77 SEM (equal to 1.96√2 × SEM) takes into account measurement error, the combined variability across the baseline and post intervention samples, and chance at the 95% confidence interval [32,33]. A measure that is not responsive at the individual level would find less than 2.5% of the sample to have an increase and 2.5% to have a decrease in their change score greater than 2.77 SEM, and therefore finding greater than this proportion would suggest WEMWBS was detecting ‘true’ change at the individual level. The SEM was calculated by multiplying the standard deviation (SD) of the baseline score by the square root of one minus the reliability of the instrument [34]. The statistic used to summarise the reliability of the instrument is either the intraclass correlation coefficient (ICC), obtained from test–retest studies, or Cronbach’s alpha [22]. As the reliability of the instrument is sample dependent, we were only able to undertake analysis on studies where item level data for WEMWBS was provided. For each study, Cronbach’s alpha was determined using the baseline WEMWBS data.

## Results

Table 1 summarises the twelve included studies: all but one recruited adults and all but three were carried out in general population samples. Table 2 shows that for 4/12 of the studies the percentage of missing data was over 50%, whilst for 5/12 studies there was no missing data. Studies included ranged in sample size from 33 to 1071, with 9/12 studies providing data on 50 or more participants. In all of the studies, fewer than 15% of participants demonstrated either a floor or ceiling effect. Baseline mean WEMWBS scores were generally lower than that found in the initial population study (mean=50.7) [13], suggesting preferential recruitment to intervention studies of those with lower mental well-being. For baseline WEMWBS scores the Shapiro-Wilk test was non-significant except in two studies (PEIP and PsyWell), for change scores the Shapiro-Wilk test was significant in the majority of the studies. However, visual examination of histograms and normality plots of both baseline and changes scores approximated to normal distribution (data available from authors upon request), suggesting the data did not violate the normality assumption.

**Table 1:** Description of included studies

**Evaluators**	**Population**	**Age**	**Sex**	**Intervention**	**Duration of intervention**
Perth and Kinross Local Authority	Unpaid carers	Adults	Mixed	Complementary therapy to support emotional health and well-being	12 weeks
Foundation for Positive Mental Health	Healthy self referred working adults	30-65	Mixed	Self help audio intervention	12 weeks
Family Links	Healthy Parents	30-40	Mixed	Group-based parenting programme	10 weeks
Body and Mind, Coventry and Warwickshire Mind	Individuals with schizophrenia, bipolar, depression and anxiety disorders	Adults	Mixed	1-1 sessions providing nutritional advice, physical activity and relaxation therapy	12 weeks
Parenting Early Intervention Pathfinder (PEIP)	Healthy parents of children with problem behaviour	Adults	Mixed	Three parenting programmes: Triple P, Incredible Years and Strengthening Families, Strengthening Communities	8-12 weeks
Recovery through Healthy Living Evaluation, Warwick Medical School	Patients with mental illness attending psychiatric day hospital.	Adults	Mixed	Recovery programmes	12 weeks
Up for it?	Healthy self referred adults	Adults	Mixed	Health and lifestyle intervention programme offering; weight management, stress management, physical activity and stop smoking.	6 weeks
Lanarkshire					
Sligo Sport and Recreation Partnership	Healthy self referred adults	40-60	Mixed	Impact of walking in promoting positive mental health	8 weeks
PsyWell RCT, Warwick Medical School	Self referred adults	Adults	Mixed	Internet based CBT skills training programme	5 weeks
Mindfulness in Schools RCT [43], Cambridge Medical School	Healthy adolescents	14-15	Male	Mindfulness training covering the principles and practice of mindfulness meditation.	4 weeks
NHS Mental Health OP clinic, Bath	Individuals with Schizophrenia	Adults	Mixed	Provision of Clozapine monitoring services by different cadres of health professionals	12 weeks
Mental Health Research Unit, Derby University	Healthy recruited adults	Adults	Mixed	Compassion computer game, involving repeatedly searching for and finding a compassionate face amongst an array of distractor faces	1 week

**Table 2:** Comparison of studies included in analysis

**Evaluators**	**Missing data (%)**	N**	**Baseline score**		**Change score**
			**Mean (SD)**	**% Floor/Ceiling**	**Mean (SD)**
Perth and Kinross Local Authority	0	85	39.3 (8.9)	1.2/1.2	10.6 (7.8)
Foundation for Positive Mental Health	50	50	44.6 (8.0)	1.0/2.0	5.7 (6.3)
Family Links	0	153	42.6 (9.4)	0.7/0.7	7.3 (8.4)*
Body and Mind, Coventry and Warwickshire Mind	9	145	40.3 (9.2)	0.6/0.6	6.2 (8.3)*
Parenting Early Intervention Pathfinder (PEIP)	50	1071	43.5 (10.4)*	0.1/0.5	7.2 (9.9)*
Recovery through Healthy Living Evaluation, Warwick Medical School	20	42	34.0 (7.7)	1.4/1.4	8.1 (11.3)*
Up for it?	62	404	42.2 (10.8)	0.2/0.2	7.0 (10.2)*
Lanarkshire					
Sligo Sport and Recreation Partnership	0	33	51.6 (9.6)	3.0/3.0	3.8 (7.7)*
PsyWell RCT, Warwick Medical School	64	557	42.5 (9.4)*	0.4/0.2	2.3 (7.7)*
Mindfulness in Schools RCT [43], Cambridge Medical School	27	78	49.7 (6.0)	0.7/0.7	1.2 (6.1)*
NHS Mental Health OP clinic, Bath	0	33	44.6 (10.7)	3.0/9.1	0.1 (9.6)*
Mental Health Research Unit, Derby University	0	61	50.4 (8.4)	1.6/3.3	−0.6 (6.5)

*P<0.05 for Shapiro-Wilk test for normality.

**available for complete case analysis.

**Figure 1:**
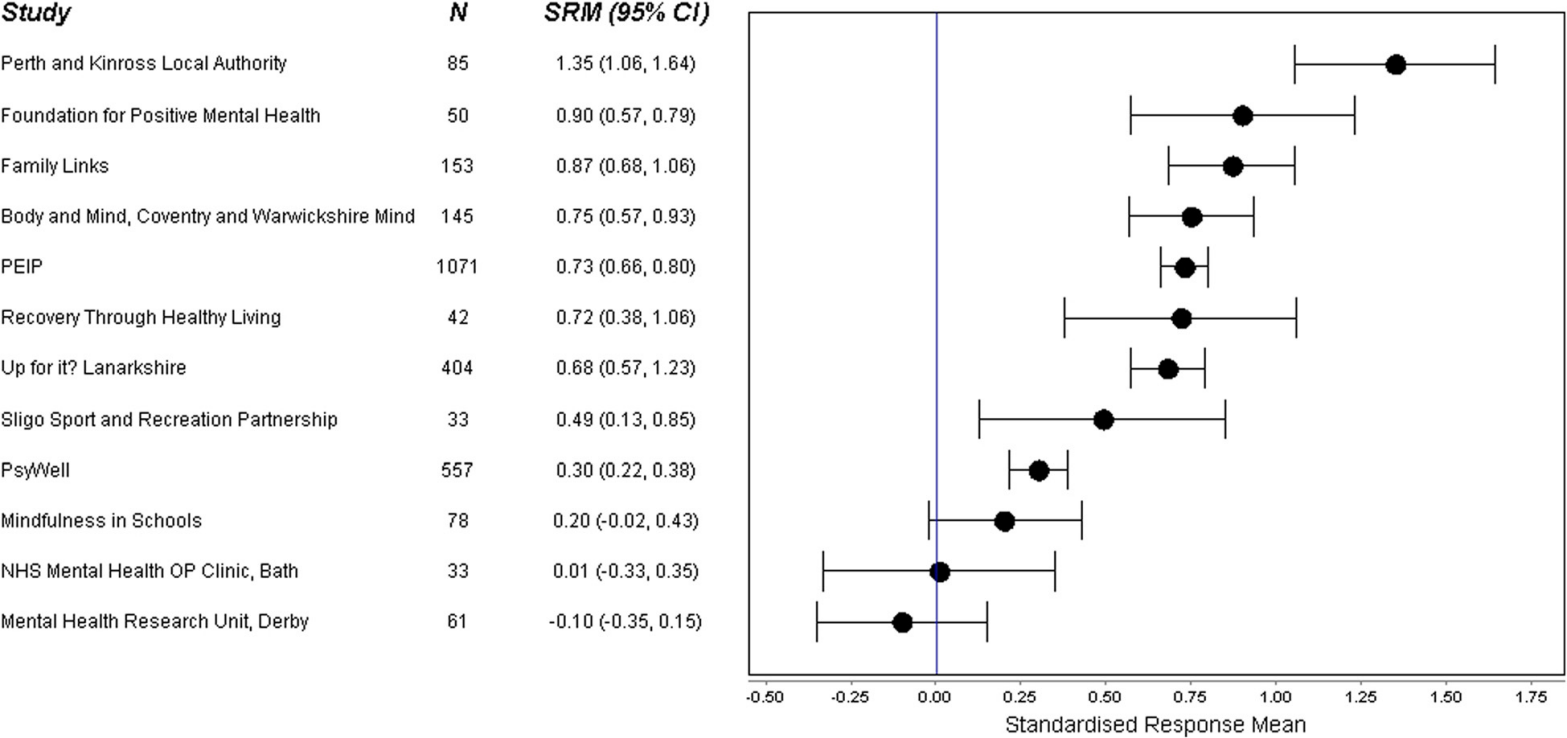
Forest Plot with Standardised response mean (SRM) for included studies.

**Figure 2:**
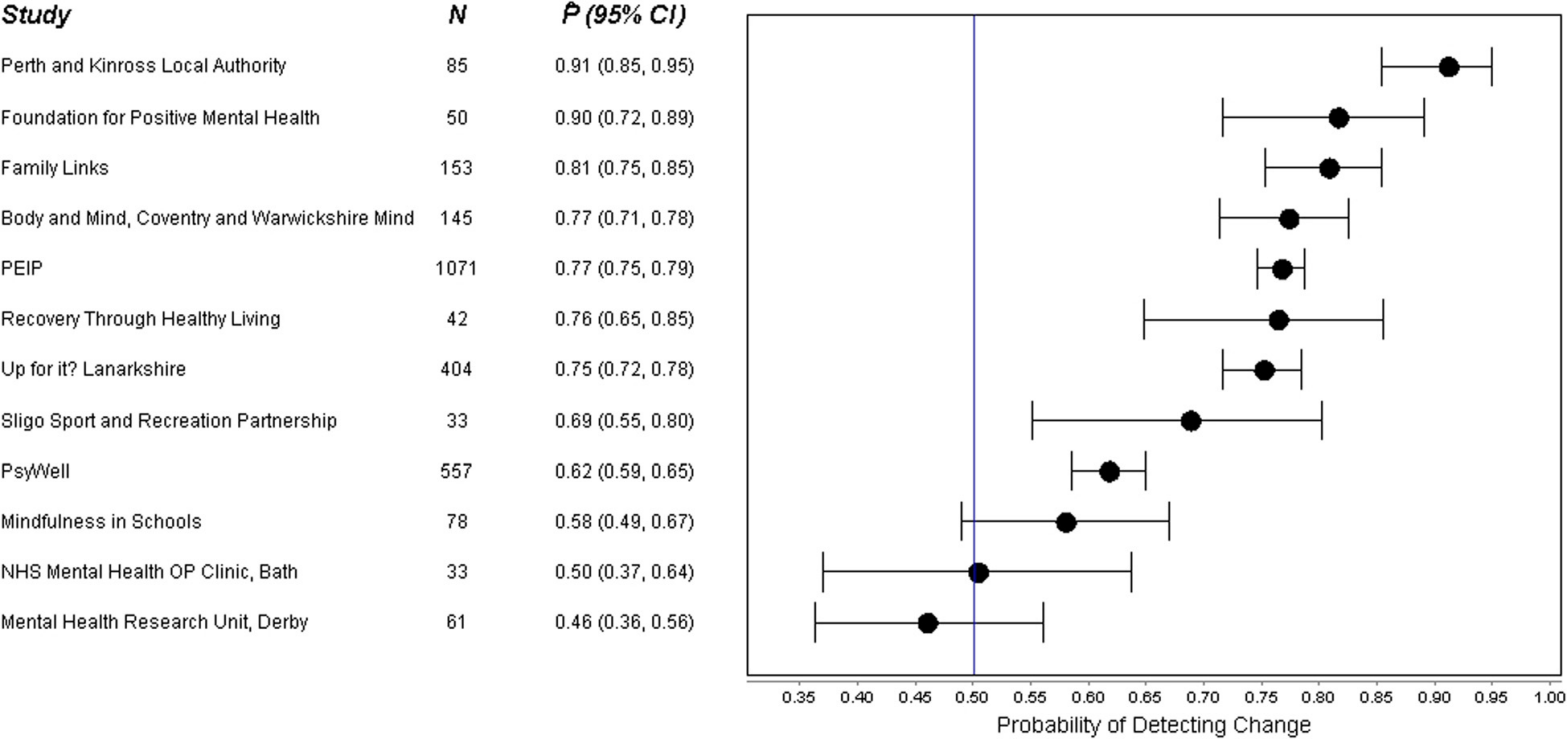
Forest Plot with Probability of detecting change for included studies.

Figure 1 shows the SRM and Figure 2 the probability of change statistic P^^^ for all twelve studies. The SRM ranged from −0.10 (95% CI: -0.35, 0.15) to 1.35 (95% CI: 1.06, 1.64), and the probability of change statistic P^^^ ranged from 0.46 (95% CI: 0.36, 0.56) to 0.91 (95% CI: 0.85, 0.95). In nine of the studies the lower limit of the 95% CI for P^^^ was greater than 0.5, whilst in six of the studies, the lower limit of the 95% CI for P^^^ was greater than 0.7. There was no evidence of any association between sample size and SRM.

Table 3 shows the findings from the individual level analysis. Of the twelve studies, item data for WEMWBS was only available for five: four undertaken in an adult population and one undertaken in an adolescent population (Mindfulness in Schools). Cronbach’s alpha for the four studies in the adult population was ≥0.864 and for the one study in the adolescent population was 0.733. The SEM for the four studies in the adult population ranged from 2.4 to 2.8, and for the one study in the adolescent population was 3.1. At the 2.77 SEM threshold 12.8% to 45.7% (lower limit of 95% CI >5.0%) of participants in the studies were found to demonstrate an increase in their score.

**Table 3:** Evaluation of individual level responsiveness

**Evaluators**	**N**	**Cronbach alpha**	**1 SEM**	**2.77 SEM**	**% Improved at 2.77 SEM (95% CI)**
Recovery through Healthy Living Evaluation, Warwick Medical School	42	0.864	2.84	7.87	45.2 (30.2, 60.3)
Parenting Early Intervention Pathfinder (PEIP)	1071	0.926	2.83	7.85	45.7 (42.7, 48.6)
PsyWell RCT, Warwick Medical School	557	0.938	2.35	6.51	27.7 (23.9, 31.4)
Mindfulness in Schools RCT [43], Cambridge Medical School*	78	0.733	3.09	8.55	12.8 (5.4, 20.2)
NHS Mental Health OP clinic, Bath	33	0.933	2.77	7.69	18.2 (5.1, 31.3)

*Study undertake in Adolescents.

SEM: Standard Error of Measurement.

CI: Confidence Interval.

## Discussion

This is the first study of which we are aware to evaluate the responsiveness of a mental well-being measure at both group and individual level. The study was possible because of the popularity and uptake of WEMWBS in evaluating interventions designed to improve mental well-being. Although WEMWBS was developed to measure mental well-being at the group or population level there has also been demand from investigators to use the scale at the individual level. It is important therefore that we found WEMWBS to be responsive at both levels. Responsiveness was independent of the type of intervention and sample size, and whilst we cannot know with certainty whether the interventions delivered in the different studies were effective, our results suggest that WEMWBS is responsive in relatively small samples. WEMWBS is likely to be responsive because it evaluates individual mental well-being across both the eudaimonic and hedonic dimensions, and is therefore more able to detect changes in their mental well-being.

When evaluating responsiveness at the group level through distribution-based approaches, the SRM is considered the most appropriate statistic [27]. However, in the literature a variety of statistics have been used to evaluate responsiveness at the group-level, including the paired *t*-test [35] and Cohen’s effect size (mean change in score by the standard deviation of the baseline score) [36]. In contrast to the paired t-test, the SRM is a sample size free statistic and therefore allowed us to compare responsiveness in studies of different sample sizes. Cohen’s effect size is dependent on between-subject variability, whilst the SRM is dependent on within-subject variability[37]. As our objective was to evaluate responsiveness of WEMWBS in detecting within-subject change we choose the SRM. Interestingly, the similarity in the standard deviation of the baseline and change WEMWBS scores in the studies evaluated means that Cohen’s effect size will be comparable to the SRM for each study.

In the majority of studies the SRM was greater than 0.5. This compares favourably to other mental illness and life satisfaction scales [38-40], generic health-related quality of life scales [41], and disease specific scales [42]. We found WEMWBS demonstrated minor floor or ceiling effects (<5%), considerably less than the 15% threshold which has been proposed [25], and to be responsive in studies undertaken in those with and without underlying mental health problems. This contrasts with mental illness scales that tend to be more responsive in populations with mental health problems [38]. Although in the majority of studies the mean baseline score was below previously reported population norms, these findings suggest that WEMWBS has the capacity to detect change in populations with both good and poor mental health, and to detect subtle improvements.

In evaluating the significance of the SRM we determined the probability of change statistic P^^^ as it provides a very intuitive interpretation of responsiveness at the group-level. A value of 0.5 suggests that if a change has occurred the instrument is as equally likely to have detected the change, as it is to have not detected the change, and therefore is not responsive. For the majority of the studies, we found the probability of change statistic P^^^ to be above 0.7, suggesting WEMWBS is responsive at the group level [27]. We assumed that in all the studies the interventions were effective at improving mental well-being. The fact that WEMWBS was not responsive at the group level in all studies could be because the interventions were not effective or because WEMWBS is not responsive to change in those populations. Only one study was undertaken in an adolescent population (Mindfulness in Schools [43]). In this study, WEMWBS was not found to be responsive. The test-retest reliability coefficient for WEMWBS in the validation study undertaken in an adolescent population (0.66) [17] was lower than the corresponding coefficient in the validation study undertaken in an adult population (0.83) [13]. It is possible that WEMWBS may not be as responsive to change in adolescents, however, further research is needed to investigate this and whether participant characteristics impact on the responsiveness of WEMWBS.

In the five studies where item level data was available, we found WEMWBS performed reasonably well in detecting change at the individual level. There is as yet no agreed consensus on what constitutes an important change at the individual level, with some suggesting that a change score greater than 1 SEM is important [34], whilst others suggest 2.77 SEM [32]. In all five studies, we found at the higher threshold of 2.77 SEM, the lower limit of the 95% confidence interval for the proportion of individuals classified as improved was greater than the 2.5% expected if WEMWBS was not responsive at the individual level. An important finding from the individual level analysis was the relatively stable Cronbach’s alpha score in adult populations. In the four studies undertaken in an adult population, the Cronbach’s alpha score was consistently high and comparable to the validation study undertaken in the adult population [13]. In the one study undertaken in the adolescent population, WEMWBS demonstrated satisfactory internal consistency [44], however, the Cronbach’s alpha score was lower than that found in the validation study undertaken in an adolescent population [17].

It has been suggested that the SEM of a measure is independent of the sample [34]. It was note-worthy to find that across the five studies for which item level data was available, the SEM was relatively comparable, suggesting that a single change in WEMWBS score could be applied to classify individuals as improved. Previous literature suggests that an improvement of 0.5 units on each item on a Likert scale would equate to an improvement deemed important by individuals [45]. This makes intuitive sense and equates to an overall change score of 7. In the studies evaluated we found a change score of 8 or more equated to statistical importance at the higher threshold of 2.77 SEM. However, a change of 3 or more units (1 SEM) in an individual’s WEMWBS score was greater than the measurement error in the majority of the studies, and thus could be interpreted as important. Further research with comparison to self-reported global ratings of change (GRC) is warranted.

Our conclusions are potentially limited, mainly as a consequence of the data used. We used data from registered users who had replied to our request to use their data, assumed that data was missing at random and only looked at change in those who had undergone an intervention. It is possible, in contravention of copyright, that there are users of WEMWBS who had not registered their use on our database. It is also possible that the reason registered users had not replied to our request was because they had not found a positive finding in their study. It is possible that missing post-intervention WEMWBS scores were not missing at random. This may potentially lead to biased estimates of treatment effect, however, our objective was to determine whether WEMWBS could detect changes in individuals’ mental well-being had they occurred. In evaluating responsiveness, it is this within-person change that is considered relevant [22]. We use distribution-based approaches to evaluate responsiveness to the exclusion of the anchor-based approaches that are increasingly favoured. Anchor-based evaluation requires a GRC. These have been associated with limitations including recall-bias, lack of validity, and are possibly insensitive to prospectively evaluated change [27,29]. Importantly, GRCs have been used in evaluating instruments measuring physical health. Whether they have construct validity in denoting improvement in mental well-being is not yet known, and therefore anchor-based approaches may not be appropriate. It is also widely acknowledged that where GRCs are not available, statistical approaches to evaluating responsiveness are valid [21,22,27,28,37]. As with any population measure, some of the changes observed may represent regression to the mean. The fact that change was observed in population groups with average as well as those with low baseline scores suggests that not all change can be attributed to this phenomenon.

## Conclusion

The relative scarcity of instruments with sufficient evidence of validity, reliability acceptability to measure mental well-being has hindered the development of policy and practice in mental health promotion. WEMWBS is a valid, reliable and acceptable measure [13], which we have demonstrated, is responsive to change in a wide variety of settings from the community settings, to schools, and psychiatric hospitals, making it suitable for use in evaluation of interventions at group and individual level. It therefore offers great potential to further research and development on mental well-being.

## Competing interests

There are no conflicts of interest regarding the contents of this article. This research would not have been possible without support from NHS Health Scotland to maintain a database of users of WEMWBS. We received no specific grant from any funding agency in the public, commercial or not-for-profit sectors for the analysis of this data.

## Authors’ contributions

HM and SSB designed the study, analysed the data and drafted the manuscript. SWP and JP aided in interpretation of data and critical revision of the manuscript. All authors contributed to final manuscript and approved the decision to submit the manuscript.
